# Diversifying Selection Underlies the Origin of Allozyme Polymorphism at the Phosphoglucose Isomerase Locus in *Tigriopus californicus*

**DOI:** 10.1371/journal.pone.0040035

**Published:** 2012-06-29

**Authors:** Sean D. Schoville, Jonathan M. Flowers, Ronald S. Burton

**Affiliations:** Marine Biology Research Division, Scripps Institution of Oceanography, University of California San Diego, La Jolla, California, United States of America; University of Arkanas, United States of America

## Abstract

The marine copepod *Tigriopus californicus* lives in intertidal rock pools along the Pacific coast, where it exhibits strong, temporally stable population genetic structure. Previous allozyme surveys have found high frequency private alleles among neighboring subpopulations, indicating that there is limited genetic exchange between populations. Here we evaluate the factors responsible for the diversification and maintenance of alleles at the phosphoglucose isomerase (*Pgi*) locus by evaluating patterns of nucleotide variation underlying previously identified allozyme polymorphism. Copepods were sampled from eleven sites throughout California and Baja California, revealing deep genetic structure among populations as well as genetic variability within populations. Evidence of recombination is limited to the sample from Pescadero and there is no support for linkage disequilibrium across the *Pgi* locus. Neutrality tests and codon-based models of substitution suggest the action of natural selection due to elevated non-synonymous substitutions at a small number of sites in *Pgi*. Two sites are identified as the charge-changing residues underlying allozyme polymorphisms in *T. californicus*. A reanalysis of allozyme variation at several focal populations, spanning a period of 26 years and over 200 generations, shows that *Pgi* alleles are maintained without notable frequency changes. Our data suggest that diversifying selection accounted for the origin of *Pgi* allozymes, while McDonald-Kreitman tests and the temporal stability of private allozyme alleles suggests that balancing selection may be involved in the maintenance of amino acid polymorphisms within populations.

## Introduction

Although there is considerable interest in quantifying adaptive variation among naturally occurring populations [1] and using this information in the management of populations [2], prospecting for adaptive variation in unknown genomes often requires considerable resources [3] or long-term experimental studies [4]. However, the advent of genomic resources and new integrative approaches has greatly expanded our ability to identify adaptive genetic variation in non-model organisms [5] and motivates a reexamination of previously identified polymorphism at functional allozyme loci. In particular, phosphoglucose isomerase (*Pgi*, E.C. 5.3.1.9, also known as glucose-6-phosphate isomerase) frequently exhibits polymorphism in natural populations and has been linked with natural selection in several taxa [6]–[10]. This has led Wheat [11] to argue that *Pgi* could be a useful candidate gene for studying adaptive genetic variation in a variety of arthropods.

The marine copepod *Tigriopus californicus* is known to have genetic polymorphism at the *Pgi* locus, with strong population divergence over a very small spatial scale [12]. For example, a single rocky outcrop at Pescadero, California, maintained a fast-migrating allele (*Pgi* ^F^) at approximately 50% in samples taken between 1978 and 1996 [13]. This allele was either absent or found at extremely low frequency at the neighboring outcrop (designated Site 10) 500 m to the south [14]. Site 10 similarly maintained a slow-migrating allele (*Pgi* ^S^) that has been recorded only intermittently at very low frequency at the Pescadero site [12]. While *Pgi* variability in each population is not exceptional, the fact that variation is maintained when local populations are subject to fluctuations in population density due to their ephemeral tidepool habitat [15] suggests that *Pgi* polymorphism might be maintained by selection.

*T. californicus* populations are among the most sharply structured of any marine organism [12], [16]. Phylogeographic studies indicate long-term divergence among populations at a small geographical scale [17], which is further emphasized by uncorrected mitochondrial DNA (mtDNA) interpopulation divergences frequently exceeding 15% [16]. Fine scale allozyme surveys have also suggested historical isolation of local subpopulations, with high frequency private alleles found in many populations [12]–[14], [18]. At migration-drift equilibrium, the average frequency of neutral private alleles is inversely related to the extent of gene flow among neighboring populations [19]. And in most cases, private alleles at several allozyme loci in *T. californicus* are restricted to narrow (i.e., <25 km) stretches of coastline and in exceptional cases are primarily restricted to individual rocky outcrops. The geographic distribution of alleles is also stable through time [13], suggesting that individual rocky outcrops throughout California are effectively closed to immigration and emigration over periods spanning hundreds of generations. While the deep genetic subdivision suggests populations of *T. californicus* are independent evolutionary lineages, laboratory tests have shown that they remain reproductively compatible [18], lacking either assortative mating or offspring inviability at the F1 stage in interpopulation crosses.

Here we characterize *Pgi* allozyme polymorphism in *T. californicus* at the DNA sequence level. We investigate whether (1) natural selection has contributed to the origin of genetic polymorphism among divergent lineages, and (2) what processes are maintaining genetic variation at the *Pgi* locus. We report DNA polymorphism data for 43 chromosomes sampled from populations throughout the southern range of the species and conduct additional allozyme surveys at populations that have been the subject of previous genetic studies. Using neutrality tests and a codon-based model of substitution, we find evidence of positive selection acting on multiple charge-changing amino acid sites. Our allozyme surveys demonstrate that allele frequencies of *Pgi* in *T. californicus* populations show stable demographic trends through a 26-year time span. In contrast to the expectation of alleles under positive selection, this temporal analysis of *Pgi* allele frequencies demonstrates stability of charge-changing polymorphisms over the course of approximately 200 generations of the *Tigriopus* lifecycle. Based on results from McDonald-Kreitman neutrality tests, we discuss how *Pgi* polymorphism is likely maintained by balancing selection in multiple independent populations of *T. californicus*.

## Methods

### Sample Collection

*T. californicus* inhabits tidepools along the supralittoral fringe of rocky intertidal habitats on the Pacific coast of North America. Animals were collected from locations throughout California and Baja California, Mexico between May, 2001 and December, 2004 and transported live to Scripps Institution of Oceanography, La Jolla, California. Copepods from Playa Altamira, Mexico, have been shown to be partially reproductively incompatible with *T. californicus* from California and we include them here as an out-group. With the exception of samples from two new sites, Mussel Rock (37° 20′ 55″ N/122° 24′ 08″W) and Pomponio Beach (37° 17′ 48″ N/122° 24′ 25″ W), collection sites are the same as those reported previously [12]–[14], [18], [20]. Although allozyme studies of Punta Morro and Playa Altamira, Mexico, were completed soon after collection, DNA analyses were based on animals maintained in the laboratory for a period of up to three years. All other samples were either prepared for molecular analysis immediately or maintained at 20° C with a 12∶12 light-dark cycle for a maximum of five days. The latter samples were carefully checked to ensure that no mortality had taken place prior to preparation for molecular analysis.

### Cloning of Phosphoglucose-isomerase

*Pgi* was amplified from cDNA using degenerate primers designed from an alignment of sequences from *Drosophila melanogaster* (Accession number: NP_523663.1), *Gryllus veletis* (AAG15513.1), *Danio rerio* (AAH44450.1), and *Homo sapiens* (AAP72966.1). Primers were designed to match seven or more amino acids which consisted of low degeneracy sites that were completely conserved among the four species in the alignment, at six different regions of the *Pgi* alignment. Total RNA was extracted with TRI Reagent (Sigma) from copepods collected from the Ocean Beach Pier collecting site, San Diego, CA. First strand cDNA synthesis was primed with an oligo(dT) primer with an extension at the 5′ end to facilitate subsequent 3′RACE. Touchdown PCR was performed with cycling conditions consisting of a 30 s 95° denaturation step, an initial annealing step of 30 s at 56° C and an extension step of 2 m at 72° C. Every 2 cycles for the first 12 cycles, the annealing temperature was stepped down by 1°C. The final 23 cycles were conducted with an annealing temperature of 50°C. A second round of PCR utilizing the same PCR primers was performed with a 50°C annealing temperature for 35 cycles with 1.0 µl of the original unpurified PCR product as template. Following two rounds of PCR, two degenerate primer pairs (5′CCIYTNATGGTNACNGARGC3’/5′TCCATRTCNCCYTGYTGRAARTA3’ and 5′TAYTTYCARCARGGNGAYATGGA3’/5′ARYTCIACNCCCCAYTGRTC3’) each yielded a single PCR product of the size expected from the original *Pgi* alignment. These products were gel purified, cloned using the pCR-4-TOPO vector (TOPO TA cloning Kit, Invitrogen), and sequenced on a MegaBace sequencer (GE Health Sciences). The section of the gene between the two original products was subsequently amplified from cDNA with *T. californicus*-specific primers. The 5′ and 3′ ends of the gene were obtained by RLM-RACE (Generacer Kit, Invitrogen).

### Allozyme Electrophoresis, PCR, and DNA Sequencing

A subset of nine samples from California and northern Baja California were examined for allozyme variation. Animals were homogenized in 10 µl of chilled buffer (0.1M Tris-Borate-EDTA, pH 8.9, with 0.12 g/ml sucrose, 10 µg/ml bromphenol blue added for loading and tracking). Following Burton [21], five µl of the homogenate was loaded directly on an acrylamide gel for allozyme electrophoresis and gels were stained for *Pgi* (NADP was replaced with NAD for use with a recombinant glucose-6-phosphate dehydrogenase coupling enzyme from *Leuconostoc mesenteroides*, E.C. 1.1.1.49, Sigma).

Some of the individuals used for allozyme electrophoresis were also included in the sequencing analysis. A 5 µl aliquot from the allozyme homogenate was prepared for PCR immediately following loading of allozyme gels. Samples were treated with five µl of Proteinase K (0.2 mg/ml) and incubated at 65°C for one hour and 85°C for 15 minutes. Samples from Pescadero (n  = 13), San Diego (n  = 14), and Santa Cruz (n  = 5) were selected randomly (i.e., independent of allozyme genotype) for sequencing to allow for estimation of population genetic parameters. In contrast, of the five alleles sequenced from Site 10, two *Pgi* ^M^/*Pgi* ^S^ heterozygotes were selected specifically to characterize the *Pgi* ^S^/*Pgi*.^89^ allele [14]. However, we were unable to unambiguously characterize the amino acid replacement responsible for this allozyme allele. Single *Pgi* ^S^/*Pgi* ^S^ and *Pgi* ^M^/*Pgi* ^M^ homozygotes from Laguna Beach were selected for sequencing based upon allozyme genotype. Single sequences were obtained from additional populations from Carpinteria, Abalone Cove, Punta Morro, and Playa Altamira populations.

All *Pgi* sequences were obtained by amplifying the entire structural gene of approximately 2.5 kb from genomic DNA with primers located in the 5′ and 3′ untranslated regions (UTR). PCR products were then cloned to determine the gametic phase of polymorphisms. Full length *Pgi* was amplified from Carpinteria and Playa Altamira samples with primers TcPGI-5′-34F and TcPGI-STOP+21R. All other samples were amplified with primers TcPGI-5′UTR-F and TcPGI-3′UTR-R (**Table S1**). Initially, at least one homozygote for *Pgi* ^F^ and *Pgi* ^M^ (i.e., *Pgi* ^1^.^05^ and *Pgi* ^1^.^00^ in previous nomenclature) allozyme alleles from San Diego and Pescadero populations were amplified and sequenced directly for characterization of charge-changing residues [14]. Subsequent PCR products were gel extracted, concentrated by ethanol precipitation, and cloned using either the pCR-4-TOPO or TOPO XL vectors (TOPO TA cloning kit, Invitrogen). Inserts were amplified with a set of four partially overlapping pairs of primers (**Table S1**) and both strands of the PCR products were sequenced on a Megabace capillary sequencer. Sequences are deposited at NCBI’s GenBank with accession numbers: JX089404-JX089454.

### Analysis of Population Structure

Sequences were edited with Sequencer version 4.5 (Genecodes, Ann Arbor, Mich.) and aligned by pairwise alignment, followed by minor adjustments to the alignment made by eye. MrBayes 3.1.2, an unrooted Bayesian method [22], was used to reconstruct the genealogical history of *Pgi* alleles. To determine which DNA substitution model would serve as an appropriate prior for gene-tree estimation, we used Akaike Information Criteria (AIC) in the program MrModeltest v.2 [23] and selected the general time reversible (GTR) model with gamma-distributed rate variation. In each of two independent MrBayes runs, four chains were sampled every 1000 steps over a total of 30 million steps. Runs were checked for convergence using Tracer [24], 10,000 samples were discarded as burnin, and a majority-rule consensus tree was estimated from the two runs.

We also estimated population structure (*F_ST_*) and migration rates (*Nm*) between regional populations, and calculated interpopulation genetic distances based on the Jukes and Cantor substitution model [25] in MEGA version 2.1 [26]. Introns and a small fragment of the 3′UTR were excluded due to alignment ambiguities among some populations and distances were calculated as net between-population means (i.e., corrected for within population distances).

### Site Frequency Tests of Recombination, Linkage Disequilibrium and Neutrality

We first tested for evidence of recombination and linkage disequilibrium using DnaSP v.5.0 [27]. The per gene recombination parameter *R* [28], equivalent to 4*Nr*, and the minimum number of recombination events *Rm* [29] were estimated for the entire dataset, as well as geographic subsets of the data. Linkage disequilibrium (LD) between pairs of polymorphic sites was estimated using the following summary statistics: average LD measured by *ZnS*, average LD between adjacent sites measured by *Za,* the difference between *Za*-*ZnS* measured by *ZZ,* LD among segregating sites measured by *B*, and LD among unique data partitions measured by *Q* [30]–[32]. Additionally, the Genetic Algorithm Recombination Detection (GARD) in Datamonkey [33] and the SiScan, MaxChi, Chimaera, and 3seq methods calculated in RDP2 [34] were used to identify recombinant alleles.

Estimation of population genetic parameters and tests of neutrality were conducted with DnaSP. We tested the assumption of neutrality at segregating sites in *Pgi* using several summary statistics, including Tajima’s *D* [35], Fu and Li’s *D_FL_* and *F* [36], the Hudson-Kreitman-Aguadé (*HKA*) statistic, and the McDonald-Kreitman (*MK*) statistic. A negative Tajima’s *D* test statistic signifies an excess of low frequency polymorphisms, as a result of positive selection or recent population expansion [35]. Under Fu and Li’s tests, negative values indicate an excess of mutation in external branches, which can arise when an advantageous allele becomes fixed or purifying selection removes deleterious alleles [36]. The HKA test examines polymorphism within species and divergence between species at two different genetic regions, testing the idea that divergence between species will be increased for a gene under positive selection. We assessed variation in *Pgi* in reference to nucleotide variation in the Rieske iron-sulfur protein, RISP [17]. The MK test examines the synonymous and non-synonymous variation within and between species, comparing the ratio of fixed substitutions to the ratio of polymorphisms. This test would support positive selection if there is an excess of non-synonymous fixed differences, and conversely supports balancing selection if there is an excess of non-synonymous polymorphism. The *D_FL_, F,* and *MK* statistics were run using the Playa Altamira sample as an out-group.

### Test for Positive Selection Based on Codon Models

Most neutrality tests examine the ratio of non-synonymous to synonymous substitutions (*ω*) averaged over the entire coding region of a gene, requiring values of *ω*>1 for evidence of positive selection. Because selection is frequently directed at a few amino acid sites of a gene, standard neutrality tests are very stringent and often fail to detect positive selection when it occurs [37]. Yang and Nielsen [37] developed an alternative approach to detecting positive selection, using a maximum-likelihood framework to examine patterns of substitution at individual codon sites. The method is implemented in a series of nested models, where more parameter-rich models allow *ω* to vary across sites and include terms for positive selection. The likelihood of the complex models are evaluated against the simpler model using a likelihood ratio test and the chi-square distribution to assess statistical significance (with degrees of freedom equal to the number of free parameters). Because this method assumes a fixed phylogeny, it is sensitive to any recombinant alleles present in the dataset [38]. We removed recombinant alleles identified in the RDP2 analysis, re-estimated the Bayesian phylogeny with Playa Altamira as an out-group, and tested for evidence of positive selection at *Pgi*. Six substitution models were evaluated, including model M0 with *ω* fixed at all sites, model M1a where sites are nearly-neutral in two site classes (*ω* = 1 or *ω*<1), the positive selection model M2a adding a third class (*ω*>1, *ω* = 1 or *ω*<1), the discrete distribution model M3 allowing *ω* to vary unconstrained among three discrete classes, the model M7 where *ω* is drawn from a beta distribution (for 0<*ω*<1), and the M8 model adding a second site class to the beta distribution model (for 0<*ω*<1, or *ω*>1) [39]–[41]. Nested models were compared to test for heterogeneous *ω*-ratios among sites (M3 vs. M0) and to test for positive selection using two model parameterizations (M2a vs. M1a and M8 vs M7). The naïve empirical Bayes (NEB) method and Bayes empirical Bayes (BEB) method were used to test the significance of *ω*-ratios at each codon position identified as positively selected under models M2a and M8.

We also implemented two similar tests that examine codon substitutions on a per site basis, the random effects likelihood (REL) model and the internal fixed effects likelihood (iFEL) model [42], [43]. The added feature of these methods is to provide additional testing for codon sites subject to purifying selection (*ω*<<1). The REL model is an extension of the Yang and Nielsen [37] method, allowing for synonymous rate variation and using empirical Bayes factors for model testing (we use a BF>40 as a cut-off for significance testing). The iFEL test implements a site by site likelihood ratio test to detect population-level adaptation by testing for selection along internal branches (we use a *p*<0.05 for significance testing). Both tests were implemented in the Datamonkey software [33].

### Temporal Analysis of Allozyme Allele Frequencies

We obtained a single-locus estimate of the effective population size change in the San Diego, Pescadero, and Site 10 populations based upon temporal changes in *Pgi* allele frequencies. The temporal samples included in the analysis were collected at different intervals between April 1978 and December 2004. We used the maximum likelihood-based approach of Beaumont [44] to estimate effective population size in a coalescent model from multiple temporal samples of allele counts in a single population. The method utilizes MCMC importance sampling to 1) estimate the harmonic mean effective population size and 2) the joint ancestral and contemporary effective population size based on allele frequency variation, assuming a model of exponential growth or decline over the temporal interval. Our focus is on the joint ancestral and contemporary effective population size, which provides an indication of demographic trends in allozyme allele classes over time. Analyses were run for each population using the default parameters (maximum iterations 100, thinning interval 10, and 0.5 proposal distribution). The number of generations between each temporal sample is required as input for the analysis. In the laboratory, the developmental time (from egg production in generation N to egg production in generation N+1) of *T. californicus* varies with temperature, averaging 25 days at 20°C and 32 days at 15°C, while is typically four months at those temperatures [45]. Natural populations of *T. californicus* reproduce continuously throughout the year with no periods of dormancy (or resting eggs) in its life cycle. Given the extensive variation in temperature experienced by natural populations, it is difficult to accurately assess generation times in the field; we conducted analyses assuming a conservative generation time of two months. We also examined allele frequency changes across years using goodness of fit *G*-tests implemented in the R software [46].

## Results

### Gene Structure and Organization

The full length cDNA contained an open reading frame of 558 codons with a putative methionine start codon and a stop codon near the 3′ end of the transcript. Comparison of the translated cDNA from the San Diego population to *Pgi* sequences from *Homo sapiens*, *Drosophila melanogaster*, and *Anopheles gambiae* revealed identical amino acids at roughly 70% of the sites. Genomic DNA sequences indicated the presence of ten exons and nine small introns between 64 and 181 base-pair (bp) long in the San Diego population. Small indel polymorphisms ranged in size from one bp to approximately 20 bp and occurred in all nine introns in interpopulation comparisons when the Playa Altamira sequence was included in the alignment. One indel restricted to this population resulted from a compound (GT)_5_(CT)_4_ short tandem repeat located in the second intron. The final alignment of 2,668 bp included the entire structural gene, with 10 exons, 9 introns, and 12 bp of the 3′UTR.

### Replacement Polymorphism and Charge-changing Residues

The *Pgi* ^M^/*Pgi* ^F^ polymorphism at Pescadero was traced to an Arg/Gln replacement polymorphism at codon position 77 in exon 3 (**Table S2**). In Pescadero, medium-migrating (*Pgi* ^M^) alleles contained an Arg at this site and fast-migrating (*Pgi* ^F^) alleles contained a Gln at this position. In contrast, the *Pgi* ^M^ and *Pgi* ^F^ allele classes in San Diego was traced to Gly and Asp residues, respectively, at codon position 66 in exon 2 (**Table S3**). In Punta Morro, Baja California, a *Pgi* ^F^ allele also contained an Asp at codon position 66 and no additional charge-changing replacements relative to the San Diego *Pgi* ^F^ allele consistent with a single mutational origin for the fast-migrating alleles in these populations. Unfortunately, we were unable to obtain a *Pgi* ^M^ allele from this location [18]. However, the widespread *Pgi* ^M^ electromorph found in southern California does not appear to be due to convergence on charge in different populations. Finally, the *Pgi* ^M^/*Pgi* ^S^ polymorphism at Laguna Beach resulted from two charge-changing replacements (Asp/Asn at position 318, Lys/Glu at codon position 463).

### Gene Tree and Population Structure

**Figure 1:**
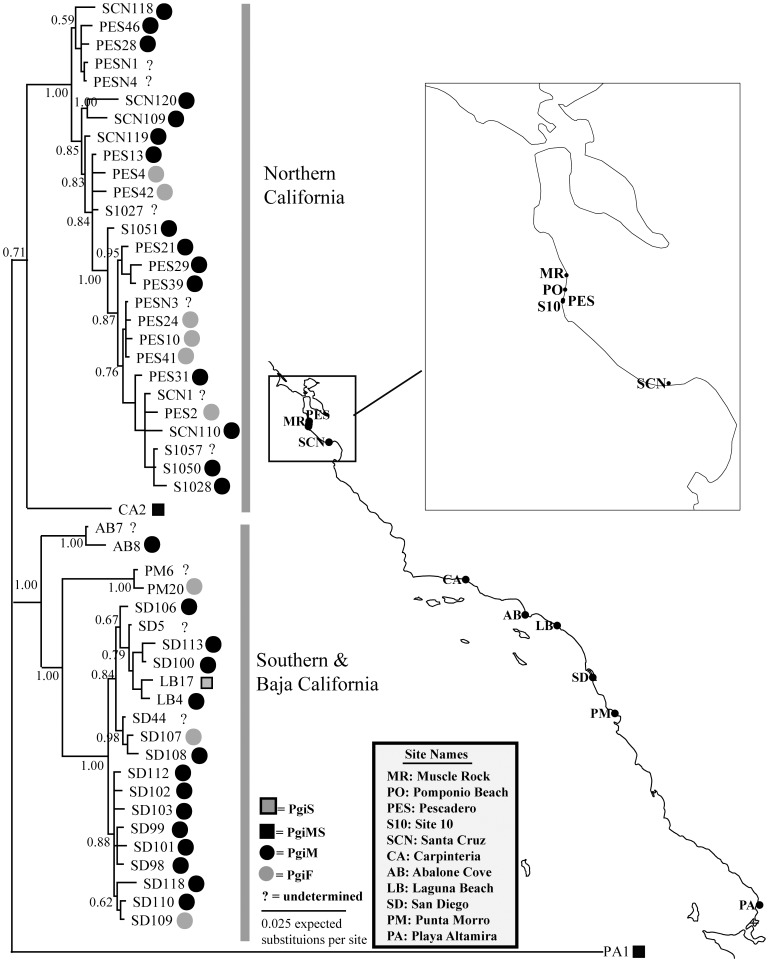
Bayesian majority-rule consensus phylogeny of *Pgi*. Sequences are labeled according to sample site, individual number, and allozyme class (slow, medium-slow, medium, fast denoted by the colored shapes).

A Bayesian gene tree analysis showed that related alleles were distributed across multiple sample sites (**Figure 1**), but a high degree of genetic structure was evident between regional populations in northern California and southern California. These populations formed reciprocally monophyletic clades (with 1.0 posterior probability branch support) separated by an average sequence divergence of 3.75% per site. The northern and southern clades did not share alleles and the alleles with charge-changing amino acids in each lineage either evolved multiple times or recombined over time. Additionally, the gene tree indicated that the partially reproductively isolated population from Playa Altamira, Baja California, Mexico was deeply divergent (∼12% average sequence divergence per site) from both the northern and southern California clades. Interpopulation distances (corrected for within population variation) at *Pgi* (**Table S4**) were comparable to other *T. californicus* nuclear genes [16], [17]. Similar to previous studies, there was evidence of multiple regionally distributed allopatric lineages with interpopulation genetic distances ranging as high as 1.5–2.5%.

### Recombination, Linkage Disequilibrium, and Neutrality Tests

Recombination was evident between segregating sites in *Pgi* as estimated from the recombination rate per gene (*R*, **Table 1**) and the minimum number of recombination events (*Rm*) in samples from Santa Cruz (SCN) and Pescadero (PES). The GARD analysis suggested that there were two break points at positions 642 and 1714 (ΔAIC_C_ 107.13), both located within coding regions. Specifically, samples PES31, PES29, PES21, PES24, PES10, PES41, and PES39 showed evidence of recombination in the RDP2 analysis. Statistical tests for linkage disequilibrium (*ZnS, Za, ZZ, B,* and *Q*) were not significant.

**Table 1:** Site frequency tests of recombination, linkage disequilibrium and neutrality at *Pgi*.

		N	*R*	*Rm*	*ZnS*	*Za*	*ZZ*	*B*	*Q*	*θ_π_*	*θ_S_*	*D*	*D_NS_*	*D_S_*	*D_FL_*	*F*	*HKA*
**All Samples**		43	0.40	48	0.19	0.23	0.04	0.04	0.07	0.03	0.050	−1.74	−2.79*	−1.52	−3.46*	−3.05*	
**Northern Clade**		24	5.30	14	0.16	0.50	0.34	0.16	0.29	0.01	0.02	−1.67	−2.50*	−0.82	−3.24*	−3.27*	
	**PESN**	13	41.80	5						0.01	0.01	−0.51	−2.05*	0.60	−1.41	−1.39	3.455
	**SCN**	5	475.00	3													1.531
**Southern Clade**		18	0.001	9	0.30	0.34	0.04	0.23	0.27	0.01	0.02	−1.89*	−2.32*	−1.63	−2.17	−2.55*	
	**SD**	14															3.467

**N** sample size; ***R*** recombination rate per gene; ***Rm*** min. no. recombination events; ***ZnS*** average LD; ***Za*** average LD between adjacent sites; ***ZZ***  =  *Za*- *ZnS*; ***B*** LD among segregating sites; ***Q*** LD among unique partitions; ***D*** Tajima’s D for all sites, non-synonymous sites (***D_NS_***) and synonymous sites (***D_S_***); ***D_FL_*** Fu and Li’s D; ***F*** Fu and Li’s F; ***HKA*** neutrality test of *Pgi* compared to RISP gene.

Neutrality tests based on site frequency spectra, including Tajima’s *D*, Fu and Li’s *D* and *F*, were used to test for selection in all samples and in separate analyses of both the northern and southern clades (**Table 2**), but only a subset of these tests were statistically significant. Tajima’s *D* was negative and significant at non-synonymous coding sites at the species-level and within populations, suggesting positive selection and/or demographic change at *Pgi*. However, Tajima’s *D* was non-significant at synonymous sites. *HKA* tests in local populations (San Diego, Pescadero, and Santa Cruz) did not provide evidence of selection. In comparisons of the northern and southern clade to the Playa Altamira out-group, McDonald-Kreitman tests showed significant excess of replacement polymorphisms, indicating balancing selection could be maintaining non-synonymous variation within each *T. californicus* clade.

**Table 2:** Site frequency tests of interpopulation genetic structure, gene flow and neutrality at *Pgi*.

	*F_st_*	*N_m_*	*S_fixed_*	*S_polymorphic_*	*NS_fixed_*	*NS_polymorphic_*	*MK p-value*
**Northern Clade vs. Playa** **Altamira**	n/a	n/a	86	39	26	47	0.000***
**Southern Clade vs. Playa** **Altamira**	n/a	n/a	83	61	23	37	0.014*
**Northern vs. Southern** **Clade**	0.68	0.12	1	95	0	82	1
**Pescadero vs. Site 10**	0.043	5.6	0	17	0	30	n/a
**Pescadero vs. Santa Cruz**	0.122	1.8	0	24	0	35	n/a
**San Diego vs. Long Beach**	0.386	0.4	2	19	0	25	0.203

***F_ST_*** among-population genetic differentiation; ***Nm*** gene flow rate between populations; ***S*** number of segregating sites; ***NS*** number of non-synonymous sites; ***MK*** McDonald-Kreitman neutrality test.

### Codon-based Test for Selection

We tested for variation in ω across codon positions in *Pgi* to determine if certain amino acid sites had elevated levels of substitution (ω>1) indicating positive selection (**Table 3**). Likelihood-ratio tests indicated that a model with discrete rate categories of ω (Model 3) fits the data significantly better (*p*<0.001) than a single rate (Model 0). Two separate model comparisons were made to look for evidence of positive selection. Based on likelihood-ratio tests, both selection models were a better fit to the data, either in comparison to a nearly neutral model (Model 2a vs. 1a, *p*<0.05) or to a model with a Beta distribution (Model 8 vs. Model 7, p<0.01). A naïve empirical Bayes (NEB) analysis indicated that positive selection is evident only at a small fraction of sites, including charge-changing amino acid shifts in the San Diego population (Asp-Gly) and the Pescadero population (Arg-Gln). All amino acid sites with elevated ω fell outside the active sites and dimer interfaces of the predicted *Pgi* protein model. The REL analysis provided additional support for positive selection at two of the same codon sites (66 and 301) with strong Bayes factor scores (>40). In addition, six codon sites were identified as under purifying selection (10, 162, 167, 182, 386, and 510) with very strong Bayes factor scores (>50). The iFEL analysis detected positive selection at three codon sites (66, 77, and 301 at *p*<0.05), notably the two charge-changing sites, and provided support for purifying selection at seven sites (10, 167, 182, 268, 388, 405, and 510 at *p*<0.05), four of which are shared with the REL analysis.

**Table 3:** Comparison of codon substitution models using likelihood-ratio tests and amino acid sites showing elevated non-synonymous substitution ratios (ω).

						Amino acid sites under selection			
PAML Model	DF	*LnL*	Nested ModelComparison	Likelihood RatioTest statistic	CodonPosition	Amino AcidChange	NEB Probω >1	BEB Probω >1	ω ± SE
M0(one-ratio)	1	−4107.2882	3 vs. 0	32.310***	45	Ser-Gln	0.676	0.643	1.241±0.594
M1a (nearly-neutral)	2	−4094.4102	2 vs. 1	6.554*	66	Asp-Gly	1.000**	0.933	1.551±0.385
M2a (selection)	4	−4091.1332	8 vs. 7	13.581**	77	Arg-Gln	0.987*	0.849	1.474±0.463
M3 (discrete)	5	−4091.1332			301	Cys-Gly-Ser-Ala	0.997**	0.890	1.513±0.423
M7 (β)	2	−4097.9314			404	Ala-Val-Met	0.837	0.713	1.331±0.529
M8 (β+ω)	4	−4091.1408			472	Thr-Glu	0.795	0.709	1.321±0.549

**DF** degrees of freedom; ***LnL*** log-likelihood; **NEB** naïve empirical Bayes test; **BEB** Bayes empirical Bayes test; **SE** standard error.

**Significant tests are indicated with an asterisk.**

### Allozyme Allele Frequencies Over Time

*Pgi* allele frequencies remained relatively stable at Pescadero, San Diego and Site 10 over a period of 26 years and *G*-tests across sampling periods were not significantly different (**Table 4**). During this time, *Pgi* genotypes rarely deviated from Hardy-Weinberg expectations, with the two exceptions occurring at Pescadero in June 1978 (*p*<0.05; *G*-test, *G*  = 10.85, *df*  = 3) and July 2004 (*p*<0.05; *G*-test, *G*  = 5.67, *df*  = 1) showing a deficit of heterozygotes. The Pescadero site maintained the widespread *Pgi* ^M^ and a private *Pgi* ^F^ allele, but the *Pgi* ^F^ allele was extremely rare at the immediately adjacent outcrop (Site 10) 500 m to the south. Instead, a private slow-migrating, *Pgi* ^S^, allele occurred at Site 10 and was not found at Pescadero. North of Pescadero, the closest suitable habitat to the north is at Pomponio Beach and Mussel Rock (4.2 km and 9.9 km from Pescadero, respectively) and these sites were nearly monomorphic for the *Pgi* ^M^ allele. An additional private allele (medium-slow) was found segregating at low frequency at these two locations, but the *Pgi* ^F^ allele was not observed. Carpinteria and Santa Cruz samples were monomorphic, while *Pgi* variation at Laguna Beach included an additional private allele and allele frequencies were comparable to a previous sample.

**Table 4:** *Pgi* allozyme allele and genotype frequencies in *Tigriopus californicus*.

			Allozyme allele frequencies								Allozyme genotype frequencies						
Site	Date^a^	N	F	M	MS	S	VS	F/F	M/F	M/M	M/MS	MS/MS	MS/S	F/S	M/S	S/S	*p*
Mussel Rock	12/28/04	38	-	.987	.013	-	–	–	–	.974	–	0.26	–	–	–	–	n.s.
Pomponio	12/28/04	36	–	.972	.028	–	–	–	–	.944	–	0.56	–	–	–	–	n.s.
Pescadero	4/78	50	.52	.48	–	–	–	.32	.40	.28	–	–	–	–	–	–	n.s.
	6/78	84	.375	.613	–	.012	–	.143	.464	.381	–	–	–	–	–	.012	.013
	8/78	104	.5	.5	–	–	–	.26	.48	.26	–	–	–	–	–	–	n.s.
	8/79	130	.485	.515	–	–	–	.223	.523	.254	–	–	–	–	–	–	n.s.
	12/2/81	30	.483	.517	–	–	–	–	–	–	–	–	–	–	–	–	n.a.
	8/27/82	83	.515	.466	–	.015	–	–	–	–	–	–	–	–	–	–	n.a.
	5/10/83	97	.459	.536	–	.005	–	.268	.381	.340	–	–	–	–	.010	–	n.s.
	8/95	66	.38	.62	–	–	–	.121	.515	.364	–	–	–	–	–	–	n.s.
	7/96	70	.51	.49	–	.014	–	.286	.414	.271	–	–	–	.029	–	–	n.s.
	7/15/04	46	.511	.489	–	–	–	.348	.326	.326	–	–	–	–	–	–	.017
	12/28/04	40	.488	.513	–	–	–	.25	.475	.275	–	–	–	–	–	–	n.s.
Site 10	8/79	70	–	.864	–	.136	–	–	–	.729	–	–	–	–	.271	–	n.s.
	8/95	66	.04	.84	–	.12	–	–	.076	.682	–	–	–	–	.242	–	n.s.
	7/15/04	48	–	.969	–	.031	–	–	–	.938	–	–	–	–	.063	–	n.s.
Santa Cruz	11/92	>50	–	1.0	–	–	–	–	–	1.0	–	–	–	–	–	–	n.s.
	2004	20	–	1.0	–	–	–	–	–	1.0	–	–	–	–	–	–	n.s
Carpinteria	2004	48	–	–	1.0	–	–	–	–	–	–	1.0	–	–	–	–	n.s.
Laguna Beach	10/92	69	.014	0.78	–	0.21	–	–	.014	.609	–	–	–	.014	.319	.043	n.a.
	2004	43	.058	.709	–	.221	0.12	–	–	–	–	–	–	–	–	–	n.a.
San Diego	10/87	>35	.11	.89	–	–	–	–	–	–	–	–	–	–	–	–	n.a.
	10/92	>50	.22	.78	–	–	–	.046	.230	.724	–	–	–	–	–	–	n.a.
	9/11/04	47	.202	.798	–	–	–	.043	.319	.638	–	–	–	–	–	–	n.s.
	9/19/04	24	.146	.854	–	–	–	.042	.208	.750	–	–	–	–	–	–	n.s.
Punta Morro	12/92	>50	.31	.69	–	–	–	–	–	–	–	–	–	–	–	–	n.a.

a Data from dates prior to 2004 were compiled from published and unpublished sources [12]–[14], [18], [20].

**Deviations of samples (of size N) from Hardy-Weinberg proportions shown as** ***p*** **-value, non-significant or not-applicable.**

**Figure 2:**
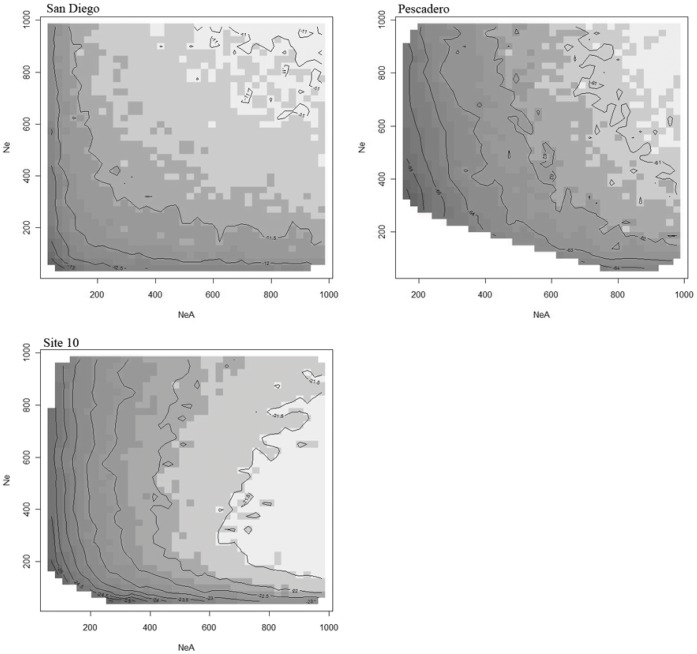
Log-likelihood surface plot of ancestral population size (*N*_e_ *A*) versus contemporary population size (*N*_e_). Estimates are based upon temporal changes in allozyme allele frequencies at *Pgi* in San Diego, Pescadero, and Site 10. The highest log-likelihood values are indicated by the white shaded contours.

We used temporal samples of allele frequencies to estimate the joint log-likelihood surface for ancestral (*N*_eA_) and contemporary population size (*N*_e_) and found no evidence of a shift in population size in either the San Diego or Pescadero populations (**Figure 2**). The stability of allele frequency estimates through time also suggests that selection did not increase or decrease the frequency of different electromorphs. In contrast, the Site 10 population showed a reduction in contemporary population size relative to ancestral population size, marked by a decline in the slow allele.

## Discussion

### Origins of Variability at the Phosphoglucose Isomerase Locus in *Tigriopus Californicus*

Based on previous observations of sharp population structure [12], [14], [18], stability of allele frequencies through time [13], and evidence of adaptive variation in other arthropods [11], we set out to examine whether allozyme polymorphism observed at *Pgi* in *Tigriopus californicus* provided evidence of natural selection. Our analysis of sequence variation focused on identifying charge-changing amino acid polymorphisms and estimating variability in previously studied populations from Baja California and California. Due to the strong phylogeographic structure and evidence of independent lineages in *Tigriopus* [17], tests based on phylogenetic as well as population-genetic methods are appropriate for evaluating evidence of natural selection. Results from these multiple statistical tests provided evidence of selection operating on amino acid polymorphisms in *Pgi*.

Site frequency tests of neutrality, including Tajima’s *D*, Fu and Li’s *D* and *F*, indicate that nucleotide variation has arisen by positive or purifying selection [35]. Statistically significant and negative values were found at both the species level and at the population level. In *Tigriopus*, there is little evidence from other genes to support population expansion in our focal populations [17], [47] and we note that these *D* values were negative despite underlying population structure. When *D* was calculated exclusively at non-synonymous and synonymous sites in *T. californicus*, only tests at non-synonymous sites were significant. While this suggested a stronger role for purifying selection, tests at synonymous sites were also negative and the lack of significance might result from reduced power due to a smaller number of sites.

Site-specific tests provided stronger evidence for positive selection acting in *Pgi*. The codon model of Yang and Nielsen [37] provided statistical support for elevated levels of non-synonymous replacements across *Pgi* codons at a small number of sites. Additional tests using REL [42] and iFEL [43] also supported positive selection at the same codon positions. We conducted these tests after removing recombinant alleles from the dataset, to avoid any bias in the phylogeny introduced by recombination [38]. Two of the selected sites were the same charge-changing amino acid polymorphisms responsible for the independently evolved *Pgi* ^F^ and *Pgi* ^M^ alleles in the northern and southern *T. californicus* clades. Linkage appeared to have decayed around these amino acid replacements, which was unexpected for a very recently derived allele because a new mutation will initially be in complete linkage disequilibrium with all polymorphisms on the chromosome on which it arises. Therefore, the decay of linkage suggested that positive selection acting on these regions is historical rather than recent.

### Maintenance of Divergent Pgi Alleles

Populations of *T. californicus* remain polymorphic for charge-changing amino acid polymorphisms and have maintained these alleles at stable frequencies for at least 100–200 generations. The population inhabiting a rocky outcrop at Pescadero, California has maintained the private *Pgi* ^F^ allele at stable frequency for a period now spanning at least 26 years. Although the local abundance of *T. californicus* frequently fluctuates over several orders of magnitude [45], the temporal changes in allele frequencies in our data are minor and could be consistent with that of a neutral allele in a population of moderate size, where genetic drift is a weak force. Although our analysis suggests that *Pgi* charge-changing amino acid polymorphisms originated as a result of positive diversifying selection, it is clear that there must be some evolutionary process acting to maintain polymorphism in local populations.

The maintenance of genetic variation in a variable environment can be advantageous to natural populations as a form of bet-hedging, insofar as the demands of natural selection require raw materials for rapid genetic change. However, the stochastic process of genetic drift, exacerbated in small or fluctuating populations, as well as selection (purifying and positive), make it difficult to sustain high levels of genetic variation. In addition to the generative role of mutation, three mechanisms act to maintain variation in natural populations. First, gene flow among structured populations can substantially increase the effective population size at neutral loci [48]. Second, recombination can act to generate variation by creating novel combinations of alleles [49]. Third, selective forces, in the form of balancing, frequency dependent or fluctuating selection [50], can actively maintain alleles in natural populations. Because these mechanisms could clearly act in concert to maintain genetic variation at a particular locus, we discuss the relative importance of each mechanism at the *Pgi* locus in *T. californicus*.

The role of gene flow in maintaining variation in *T. californicus* seems quite limited. Populations at adjacent rock-pools can be distinguished by high frequency private alleles, suggesting low rates of gene exchange [14], [19]. In a survey of populations inhabiting narrow (i.e., <25 km) stretches of coastline in central California, Burton and Feldman [14] reported high-frequency private alleles at all five allozyme loci examined (*Got* ^1.07^ at Monterey (30%), *Pgm* ^1.07^ at Capitola (25%), *Pgi* ^F^ at Pescadero (48%), *Gpt* ^1.06^ at Santa Cruz (25%), *Got* ^1.03^ at Bodega Bay (50%), and *Est*.^97^ at Moss Beach (30%)). Numerous low frequency polymorphisms are also restricted in distribution. As a result estimated rates of interpopulation gene flow are very low [12], [17] in *T. californicus*.

Recombination can act to generate allelic variation and has been suggested to play an important supporting role in maintaining adaptive polymorphisms in *Pgi* in *Colias* butterflies [51]. In our dataset, recombinant alleles were detected in the northern California Pescadero population. At a neutral locus, recombination has to be quite high (r ≥10µ) and the population size quite large (4Nµ ≥2) for recombination to have a significant effect on standing levels of genetic variation [52]. We currently lack independent estimates of either the recombination rate or the effective population size of local populations of *T. californicus*; however, we would expect to see evidence of recombination in the San Diego and Santa Cruz populations if it was acting as a general mechanism in generating *Pgi* polymorphism throughout the range of *T. californicus*.

Balancing selection remains the most probable explanation for the maintenance of variation in *T. californicus*. McDonald-Kreitman tests of both the northern and southern clades with Playa Altamira as an out-group indicate balancing selection due to a statistically significant excess of replacement polymorphisms. The preservation of polymorphism at the charge-changing sites, when nearby sites are under negative selection, is also consistent with balancing selection. Several statistical tests provide evidence of purifying selection acting on other coding regions of *Pgi*, including Tajima’s *D* and the REL and iFEL codon-based tests.

Balancing selection has also been implicated in studies of *Pgi* in two intertidal amphipods, two butterflies and a cricket [6], [8]–[10]. The amphipod species in the genus *Gammarus* are notable for their similarity to *T. californicus*, in terms of life history, habitat preference and multigenerational maintenance of *Pgi* allozyme variation. Patarnello and Battaglia [10] have further shown that clear fitness tradeoffs exist for particular *Pgi* genotypes and temperature conditions. Similar efforts to characterize the fitness of different genotypes in an ecological context will be needed to provide stronger evidence on whether balancing selection is acting to maintain variation at *Pgi* in *T. californicus*.

## Supporting Information

Table S1 **Oligonucleotide primers used for amplification and sequencing of** ***Tigriopus californicus*** **phosphoglucose isomerase (** ***Pgi*** **).**
(PDF)

Table S2 **Non-singleton polymorphisms from three sites in northern California.** The polymorphism responsible for the *Pgi* ^F^ allele is indicated in bold. The boxed region indicates the haplotype block that is conserved in all *Pgi* ^F^ alleles. The electrophoretic allele class, *Pgi* ^F^ or *Pgi* ^M^, of each sequence is indicated by an M or an F next to the sample ID. Sample abbreviation are PES  =  Pescadero, S10 =  Site 10, and SCN  =  Santa Cruz. Singleton sites have been omitted from the alignment. Numbers at the top of the table are the positions in the global alignment of all nine populations in this study.
(PDF)

Table S3 **Non-singleton polymorphisms from San Diego (SD) and Laguna Beach (LB).** The polymorphism responsible for the *Pgi* ^F^ allele is indicated in bold. The electrophoretic allele class, *Pgi* ^F^, *Pgi* ^M^ or *Pgi* ^S^, of each sequence is indicated by an F, M, or S next to the sample ID. Singleton sites have been omitted from the alignment. Numbers at the top of the table are the positions in the global alignment of all populations.
(PDF)

Table S4 **Inter-population distances at** ***Pgi*** **based upon coding regions corrected by the method of Jukes and Cantor** [**25**] **.** Distances are net between-population means (i.e., corrected for within population distances).
(PDF)
