# Supplementary material for: Diversifying Selection Underlies the Origin of Allozyme Polymorphism at the Phosphoglucose Isomerase Locus in Tigriopus californicus
Source: PLoS One. 2012 Jun 29;7(6):e40035. doi: 10.1371/journal.pone.0040035 (PMC3386920; doi:10.1371/journal.pone.0040035)
Supplement: Table S1 — Oligonucleotide primers used for amplification and sequencing of Tigriopus californicus phosphoglucose isomerase ( Pgi ). (PDF) [file pone.0040035.s001.pdf]

Schoville, S.D., J.M. Flowers, and R.S. Burton. 2012. Diversifying selection underlies the origin of allozyme polymorphism at the phosphoglucose isomerase locus in *Tigriopus californicus*

Table S1. Oligonucleotide primers used for amplification and sequencing of *Tigriopus californicus* phosphoglucose isomerase (*Pgi*).

Primer	Sequence
TcPGI-3'UTR-R	AACTAGCGCCATAACGAATAC
TcPGI-5'UTR-F	TCCCGACGAGCAAAATAGCA
TcPGI-0.5-R	GCATCACATCCTTGCCATCCAC
TcPGI-0.5-F	CCAGAAAATCAATTTCACCGAG
TcPGI-2.25-R	CCAATCCCAAAACTCGAACAT
TcPGI-2.25-F	CCTCTGCAGTGGCGAAACAC
TcPGI-3.5-R	CAAGAAGTTACACAGAAGGAG
PA-PGI-2.25F	CCGCCGCGGTGGCGAAACAC
PA-PGI-3.5R	CTAAAATGACCGTTGTGGGTCG
TcPGI-5'-34F	GCTGCAGACGAAATACACTCTC
TcPGI-STOP+21R	AAGTCAAGTCTTGGCGAGTAA
