# Supplementary material for: Diversifying Selection Underlies the Origin of Allozyme Polymorphism at the Phosphoglucose Isomerase Locus in Tigriopus californicus
Source: PLoS One. 2012 Jun 29;7(6):e40035. doi: 10.1371/journal.pone.0040035 (PMC3386920; doi:10.1371/journal.pone.0040035)
Supplement: Table S2 — Non-singleton polymorphisms from three sites in northern California. The polymorphism responsible for the Pgi F allele is indicated in bold. The boxed region indicates the haplotype block that is conserved in all Pgi F alleles. The electrophoretic allele class, Pgi F or Pgi M, of each sequence is indicated by an M or an F next to the sample ID. Sample abbreviation are PES = Pescadero, S10 = Site 10, and SCN = Santa Cruz. Singleton sites have been omitted from the alignment. Numbers at the top of the table are the positions in the global alignment of all nine populations in this study. (PDF) [file pone.0040035.s002.pdf]

Schoville, S.D., J.M. Flowers, and R.S. Burton. 2012. Diversifying selection underlies the origin of allozyme polymorphism at the phosphoglucose isomerase locus in *Tigriopus californicus*

Table S2. Non-singleton polymorphisms from three sites in northern California. The polymorphism responsible for the *Pgi*^{F} allele is indicated in bold. The boxed region indicates the haplotype block that is conserved in all *Pgi*^{F} alleles. The electrophoretic allele class, *Pgi*^{F} or *Pgi*^{M}, of each sequence is indicated by an M or an F next to the sample ID. Sample abbreviation are PES = Pescadero, S10 = Site 10, and SCN = Santa Cruz. Singleton sites have been omitted from the alignment. Numbers at the top of the table are the positions in the global alignment of all nine populations in this study.

ID	Polymorphic sites			
		111	1111111111	1111222222
	122333556	7788999012	2566777778	8999013345
	3634444689	2967138861	2158122995	5228691503
	0562056686	6215150282	4824518693	6271908582
PES39M	AGACTCTGCA	ATCAAGTTTT	CCATGTCTCA	TGGAGCCTCT
PES21M	....	....	....	......TAAC
PES31M	....ATC.GG	GGTG.C.C..	....	......TAAC
PES29M	....	....	....	....
PES28M	GACGA...GG	GGTG.C.C..	.T.CACACT.	.AAT.TTAAC
PES46M	GACGA...GG	GGTG.C.C..	...CACACTT	GAAT.TTAAC
PES13M	....ATC...	.......CC.	.T.CACACTT	GAAT.TTAAC
PES4F	....ATC**A**..	.......CC.	.T.CACACTT	GAAT.TTAAC
PES24F	....ATC**A**..	.......C..	....	......TAAC
PES41F	....ATC**A**..	.......C..	....	......TAAC
PES10F	....ATC**A**..	.......C..	....	......TAAC
PES2F	GA..ATC**A**..	GGTG.C.C..	....	....
PES42F	....ATC**A**..	.......C..	.TGCACACTT	GAAT..TAAC
S1050M	.ACGA...GG	GGTG.C.C..	....	....T.....
S1051M	....	.......CC.	.T.CACA...	......TAAC
S1057*	.ACGA...GG	GGTG.C.C..	..G.......	....T.....
S1027*	.A..ATC...	.......C..	.T.CACACTT	GAAT.TTAAC
S1028M	.ACGA...GG	GGTG.C.C..	....	....T.....
SCN109M	.A..ATC.GG	GGT.GCGCCC	GT.CACACTT	GAAT.TTAAC
SCN119M	....ATC.GG	GG.....CC.	.T.CACACTT	GAAT.TTAAC
SCN120M	....ATC.GG	GG...CGCCC	GT.C.CA..T	GAAT.A...C
SCN110M	.A..ATC.GG	.GT.GC.C..	....	C
SCN118M	...G....GG	GGTG.C.C..	...CACACTT	.AAT.T...C

*Samples from Site 10, S10 27 and 57, were *Pgi*^{M}/*Pgi*^{S} heterozygotes, but the *Pgi*^{S} allele class could not be unambiguously characterized at the sequence level.
