# Supplementary material for: Diversifying Selection Underlies the Origin of Allozyme Polymorphism at the Phosphoglucose Isomerase Locus in Tigriopus californicus
Source: PLoS One. 2012 Jun 29;7(6):e40035. doi: 10.1371/journal.pone.0040035 (PMC3386920; doi:10.1371/journal.pone.0040035)
Supplement: Table S3 — Non-singleton polymorphisms from San Diego (SD) and Laguna Beach (LB). The polymorphism responsible for the Pgi F allele is indicated in bold. The electrophoretic allele class, Pgi F, Pgi M or Pgi S, of each sequence is indicated by an F, M, or S next to the sample ID. Singleton sites have been omitted from the alignment. Numbers at the top of the table are the positions in the global alignment of all populations. (PDF) [file pone.0040035.s003.pdf]

Schoville, S.D., J.M. Flowers, and R.S. Burton. 2012. Diversifying selection underlies the origin of allozyme polymorphism at the phosphoglucose isomerase locus in *Tigriopus californicus*

Table S3. Non-singleton polymorphisms from San Diego (SD) and Laguna Beach (LB). The polymorphism responsible for the *Pgi*^{F} allele is indicated in bold. The electrophoretic allele class, *Pgi*^{F}, *Pgi*^{M} or *Pgi*^{S}, of each sequence is indicated by an F, M, or S next to the sample ID. Singleton sites have been omitted from the alignment. Numbers at the top of the table are the positions in the global alignment of all populations.

ID	Polymorphic sites		
		11111111	111122222
	34444455	8801334557	789933345
	2671778849	2316126030	704714626
	1329781211	2719193602	520784525
SD98M	GCAGCTCAGG	GAAGGTTTCA	TGACCTATC
SD99M	....	....	....
SD100M	ATC.GAGGAC	TG.CACCA.G	.TG...GCA
SD101M	....	....	....
SD102M	....	....	........A
SD103M	....	....	......GCA
SD106M	AT......A.	TG.CACCA.G	........A
SD107F	...**A**......	TG.CACCAT.	.TG...GCA
SD108M	....	...CACCAT.	.TG...GCA
SD109F	AT.**A**......	....	........A
SD110M	AT........	....	........A
SD112M	....	....	........A
SD113M	ATC.GAGGAC	TGGCACCA.G	.TG...GCA
SD118M	A.C.GAGGA.	..G.......	........G
LB4M	..C.GAGGA.	TG.CACCA.G	G.GTTG..A
LB17S	..C.GAGGA.	T..CACCA.G	G.GTTG..A
