# Supplementary material for: Diversifying Selection Underlies the Origin of Allozyme Polymorphism at the Phosphoglucose Isomerase Locus in Tigriopus californicus
Source: PLoS One. 2012 Jun 29;7(6):e40035. doi: 10.1371/journal.pone.0040035 (PMC3386920; doi:10.1371/journal.pone.0040035)
Supplement: Table S4 — Inter-population distances at Pgi based upon coding regions corrected by the method of Jukes and Cantor [25] . Distances are net between-population means (i.e., corrected for within population distances). (PDF) [file pone.0040035.s004.pdf]

Schoville, S.D., J.M. Flowers, and R.S. Burton. 2012. Diversifying selection underlies the origin of allozyme polymorphism at the phosphoglucose isomerase locus in *Tigriopus californicus*

Table S4. Inter-population distances at *Pgi* based upon coding regions corrected by the method of Jukes and Cantor (1969). Distances are net between-population means (i.e., corrected for within population distances).

	PA	PM	SD	LB	AB	CA	SCN	PES
PA	-							
PM	0.084	-						
SD	0.082	0.021	-					
LB	0.083	0.023	0.003	-				
AB	0.083	0.026	0.019	0.02	-			
CA	0.080	0.024	0.02	0.019	0.02	-		
SCN	0.078	0.02	0.015	0.015	0.016	0.014	-	
PES	0.080	0.02	0.016	0.015	0.017	0.015	0.002	-
